# Restaging after neoadjuvant FOLFIRINOX for localized pancreatic cancer: a clinical calculator from the Trans-Atlantic Pancreatic Surgery consortium

**DOI:** 10.1093/jnci/djag024

**Published:** 2026-03-17

**Authors:** Esther N Dekker, David van Klaveren, Eva M M Verkolf, Roeland F de Wilde, Marc G Besselink, Eileen M O’Reilly, Alessandro Paniccia, Alice C Wei, Amer H Zureikat, Laura R Prakash, Matthew H G Katz, Ching-Wei D Tzeng, Bas Groot Koerkamp, Trans-Atlantic Pancreatic Surgery Consortium, Matthew H G Katz, Ching-Wei D Tzeng, Bas Groot Koerkamp, Alessandro Paniccia, Alice C Wei, Amer H Zureikat, Marc G Besselink, Esther N Dekker, Eva M M Verkolf, Laura R Prakash

**Affiliations:** Department of Surgery, Erasmus MC Cancer Institute, Rotterdam, the Netherlands; Department of Public Health, Center for Medical Decision Making, Erasmus MC, Rotterdam, the Netherlands; Department of Surgery, Erasmus MC Cancer Institute, Rotterdam, the Netherlands; Department of Surgery, Erasmus MC Cancer Institute, Rotterdam, the Netherlands; Department of Surgery, Amsterdam UMC, University of Amsterdam, Amsterdam, the Netherlands; Cancer Center Amsterdam, Amsterdam, the Netherlands; Department of Medicine, Memorial Sloan Kettering Cancer Center, New York, NY, United States; Division of Surgical Oncology, University of Pittsburgh Medical Center, Pittsburgh, PA, United States; Department of Surgery, Memorial Sloan Kettering Cancer Center, New York, NY, United States; Division of Surgical Oncology, University of Pittsburgh Medical Center, Pittsburgh, PA, United States; Department of Surgical Oncology, Division of Surgery, The University of Texas MD Anderson Cancer Center, Houston, TX, United States; Department of Surgical Oncology, Division of Surgery, The University of Texas MD Anderson Cancer Center, Houston, TX, United States; Department of Surgical Oncology, Division of Surgery, The University of Texas MD Anderson Cancer Center, Houston, TX, United States; Department of Surgery, Erasmus MC Cancer Institute, Rotterdam, the Netherlands

## Abstract

**Background:**

Restaging after neoadjuvant chemotherapy aims to assess treatment response, revise prognosis, and guide further management. This study investigated independent prognostic factors for overall survival after restaging in patients with localized pancreatic adenocarcinoma.

**Methods:**

In this retrospective international study, consecutive patients with localized pancreatic adenocarcinoma who received at least 1 cycle of (m)FOLFIRINOX as first-line therapy were identified. Multivariable Cox regression analysis was performed with a web-based calculator to predict individualized overall survival.

**Results:**

The Trans-Atlantic Pancreatic Surgery cohort included 2338 patients with localized pancreatic adenocarcinoma of whom 22.6% were potentially resectable, 30.7% borderline resectable, and 46.7% locally advanced at initial staging. Several baseline characteristics remained independent prognostic factors for overall survival after restaging: borderline resectable (hazard ratio [HR] = 1.31, 95% confidence interval [CI] = 1.14 to 1.51), locally advanced (HR = 1.78, 95% CI = 1.55 to 2.05), body or tail tumor (HR = 0.79, 95% CI = 0.68 to 0.90), and baseline World Health Organization performance status of 1 (HR = 1.18, 95% CI = 1.07 to 1.30) or at least 2 (HR = 1.54, 95% CI = 1.20 to 1.98). Additional independent factors were metastatic disease at restaging (HR = 1.57, 95% CI = 1.31 to 1.87), postinduction carbohydrate antigen 19-9 (HR = 1.47, 95% CI = 1.37 to 1.57), Δcarbohydrate antigen 19-9 (HR = 0.83, 95% CI = 0.77 to 0.89), postinduction tumor size (HR = 1.22, 95% CI = 1.11 to 1.35), and Δtumor size (HR = 0.92, 95% CI = 0.87 to 0.98). Patients were stratified into 4 risk groups, with 3-year overall survival after restaging ranging from 6.0% to 65.8%.

**Conclusion:**

Survival at restaging after neoadjuvant chemotherapy of patients with localized pancreatic adenocarcinoma is determined by 8 patient, tumor, and treatment response characteristics. A web-based calculator can inform clinicians and patients about individualized prognosis and guide further management.

## Introduction

Pancreatic ductal adenocarcinoma is a highly lethal disease and is projected to become the second leading cause of cancer-related death by 2030.^1^ Cancer staging at the time of diagnosis plays a pivotal role in oncology, serving as the cornerstone for informing patients about prognosis and subsequent shared decision making.^2-4^ In clinical practice, localized pancreatic adenocarcinoma is classified into potentially resectable, borderline resectable, and locally advanced disease, based on the extent of tumor abutment to surrounding vasculature. This anatomical staging has evolved into the clinical ABC staging system, considering not only anatomy (A) but also biology (B; eg, carbohydrate antigen 19-9 [CA19-9]), and condition (C; ie, performance status).^5^^,^^6^ This ABC staging outperformed the TNM classification for classifying patients with similar prognosis.

The use of neoadjuvant therapy as an initial therapy for localized (ie, nonmetastatic) pancreatic adenocarcinoma is increasing. Fluorouracil, leucovorin, irinotecan, and oxaliplatin (FOLFIRINOX) and gemcitabine-based therapies are the preferred regimens.^7^^,^^8^ After initial therapy, restaging aims to assess treatment response, revise prognosis, and guide further management.^2^^,^^9^ Restaging has primarily been anatomical, focusing on the occurrence of distant metastases and local response. However, other anatomical, biological, and conditional factors can improve restaging.^10^

The first aim of this study was to identify independent prognostic factors for overall survival at the time of restaging in patients with localized pancreatic adenocarcinoma who started treatment with neoadjuvant FOLFIRINOX. The second aim was to predict overall survival after restaging for individual patients.

## Methods

### Study design and patients

This is a retrospective cohort study conducted within the Trans-Atlantic Pancreatic Surgery (TAPS) Consortium including all consecutive patients with localized pancreatic adenocarcinoma between January 1, 2012, and December 31, 2022. All patients were treated with at least 1 cycle of (modified) (m)FOLFIRINOX as their initial therapy. The TAPS Consortium comprises 5 high-volume pancreatic adenocarcinoma referral centers. Three are located in the United States (Memorial Sloan Kettering Cancer Center, University of Pittsburgh Medical Center, and University of Texas MD Anderson Cancer Center) and 2 in the Netherlands (Amsterdam UMC and Erasmus MC Cancer Institute). Previous publications have provided details about the TAPS Consortium’s legal structure and data collection methods.^11^^,^^12^ All participating centers obtained ethical approval from their respective institutional review boards, with informed consent requirements waived. This study adhered to the Strengthening Reporting of Observational Studies in Epidemiology guidelines for observational study reporting.^13^

### Data collection, variable definitions, and study outcomes

Patient, tumor, treatment, and outcome data were obtained from the TAPS cohort database. The initial anatomical staging was established based on tumor abutment of surrounding vessels at diagnosis to distinguish patients with potentially resectable, borderline resectable, and locally advanced pancreatic adenocarcinoma. This was assessed on radiographic imaging by specialized multidisciplinary teams comprising radiologists and hepatopancreatobiliary surgeons. The MD Anderson Cancer Center Clinical Classification System was used by MD Anderson Cancer Center, while the other centers used the National Comprehensive Cancer Network criteria.^6^ Several baseline risk factors previously associated with prognosis were considered, including tumor location (head vs body or tail, ie, left-sided), tumor size, patient age, World Health Organization performance status, and CA19-9 levels. The definitions of the prognostic factors have been published previously.^11^ On completion of neoadjuvant systemic chemotherapy, restaging was performed using cross-sectional imaging to rule out distant metastases, determine vessel abutment, reassess tumor size, and posttreatment serum CA19-9. The decision to proceed with additional treatment (eg, radiotherapy or surgical resection) was made by each center’s multidisciplinary team, accounting for the ABC criteria and shared decision making. All prognostic factors were assessed at baseline and/or after restaging. The primary outcome was overall survival after restaging (ie, rather than after tissue initial diagnosis), defined as the time from the date of restaging following initial therapy with (m)FOLFIRINOX to the date of death or last follow-up on June 26, 2024. Censoring was applied for patients who were alive at the time of last contact.

### Statistical analysis

Baseline characteristics were presented as frequencies (percentages) for categorical variables or as median with the range for continuous variables. The probability of survival over time was calculated using the Kaplan–Meier method. Univariable and multivariable Cox regression analyses were used to determine which variables predict survival after restaging. In the univariable and multivariable Cox model, all continuous predictors were included as restricted cubic splines (3 knots).^14^ All variables with a *P* value less than .05 in univariable analysis were included in multivariable Cox regression analysis. Associations were expressed as hazard ratios (HRs) with 95% confidence intervals (CIs). Continuous variables were modeled using restricted cubic splines, and hazard ratios were expressed per IQR, comparing the 75th with the 25th percentile. Missing values were imputed using multivariate imputation by chained equations implemented via the *mice* package in R.^15^ The final model developed using the independent prognostic factors for overall survival after restaging was integrated into an interactive web-based application using the R package Shiny. (The calculator can be accessed via pancreascalculator.com.) Patients were stratified into 4 risk groups based on their predicted 3-year overall survival probability derived from the final multivariable Cox model. The predicted probabilities were averaged across imputations for each patient, after which thresholds were applied to define the risk categories: very unfavorable (≤15%), unfavorable (15%-30%), intermediate (30%-50%), and favorable (>50%). Kaplan–Meier survival curves were then constructed to visualize the observed overall survival across these risk groups. The prognostic contribution of each predictor was quantified using partial Wald χ^2^ statistics from the multiply imputed multivariable Cox model, expressed as χ^2^ minus degrees of freedom to account for the number of parameters per predictor (eg, nonlinear spline terms) (Figure S3). Separate Kaplan–Meier survival curves were constructed for patients who did and did not undergo resection, for descriptive purposes only. No log-rank test was performed, as these groups were not comparable. To assess the generalizability of the model across different centers, a leave-one-center-out cross-validation was performed by consecutively fitting the model with data from 4 centers of the cohort and validating the model against data from the center that was left out.^16^ Statistical significance was defined as a *P* value less than .05. Statistical analyses were performed using R version 4.5.0.^17^

## Results

### Patient and tumor characteristics

Overall, 2338 patients diagnosed with localized pancreatic adenocarcinoma between 2012 and 2022 were included from the TAPS database. Most patients were male (54.3%), and the median age at diagnosis was 64 years (IQR = 57-69 years). Based on radiographic imaging, 22.6% of the patients were classified as potentially resectable, 30.7% as borderline resectable, and 46.7% as locally advanced disease. Most patients had a performance status of 0 (42.9%) or 1 (53.6%). The median baseline CA19-9 was 202.9 U/mL (IQR = 44.0-748.4 U/mL), the median baseline carcinoembryonic antigen was 3.7 U/mL (IQR = 2.1-7.1 U/mL), and the median baseline tumor size was 35.0 mm (IQR = 27.0-45.0 mm) (Table 1).

**Table 1.** Patient characteristics and outcomes

Baseline characteristics^a^	Entire cohort (*N* = 2338)
Male sex, No. (%)	1269 (54.3)
Age, median (IQR), y	64 (57-69)
Radiographic tumor stage, No. (%)	
Potentially resectable	527 (22.6)
Borderline resectable	715 (30.7)
Locally advanced	1088 (46.7)
Baseline CA19-9, median (IQR), U/mL	202.9 (44.0-748.4)
Baseline CEA, median (IQR), U/mL	3.7 (2.1-7.1)
Baseline World Health Organization performance status, No. (%)	
0	999 (42.9)
1	1249 (53.6)
2-4	81 (3.5)
Body mass index, median (IQR), kg/m^2^	26.1 (23.2-29.2)
Tumor size baseline, median (IQR), mm	35.0 (27.0-45.0)
Tumor location in pancreas, No. (%)	
Head or uncinate	1584 (67.9)
Neck or proximal body	369 (15.8)
Distal body or tail	380 (16.3)
Restaging characteristics^a^	
Metastatic disease at restaging, No. (%)	175 (7.5)
Post-induction CA19-9, median (IQR), U/mL	66.7 (23.0-239.0)
ΔCA19-9, median (IQR)^b^	59.4 (0-348.0)
Post-induction CEA, median (IQR), U/mL	4.2 (2.7-6.7)
Post-induction tumor size, median (IQR), mm	30.0 (23.0-40.0)
ΔTumor size, median (IQR),^c^ mm	4.0 (-1.0 to 11.0)
Treatment characteristics^a^	
No. of cycles, median (IQR)^d^	6 (4-8)
Chemotherapy switch, No. (%)	357 (15.4)
Radiotherapy,^e^ No. (%)	1045 (45.3)
Surgical exploration, No. (%)	1125 (48.7)
Resection, No. (%)	989 (42.4)
Surgical procedure,^f^ No. (%)	
Pancreatoduodenectomy	742 (76.7)
Left pancreatectomy	183 (18.9)
Central pancreatectomy	23 (2.4)
Total pancreatectomy	19 (2.0)
Adjuvant chemotherapy,^f^ No. (%)	604 (61.5)
Pathological outcomes,^a^^,^^f^ No.	989
Resection margin status,^g^ R0, No. (%)	627 (70.0)
Tumor differentiation, No. (%)	
Well, G1	27 (3.1)
Moderate, G2	582 (66.9)
Poor, G3	261 (30.0)
T stage,^h^ No. (%)	
ypT0	38 (4.5)
ypT1-2	633 (75.0)
ypT3-4	173 (20.5)
N stage,^h^ No. (%)	
ypN0	424 (43.8)
ypN1	338 (34.9)
ypN2	206 (21.3)
Perineural invasion, No. (%)	753 (78.5)
Lymphovascular invasion, No. (%)	573 (60.3)
Survival outcomes (*n* = 2338)	
Overall survival after restaging, median (95% CI), months	17.2 (16.1 to 18.3)
Overall survival after restaging, % (95% CI)	
1 y	64.0 (62.0 to 66.1)
3 y	26.4 (24.5 to 28.4)
5 y	15.7 (14.0 to 17.5)

Abbreviations: CA19-9 = carbohydrate antigen 19-9; CEA = carcinoembryonic antigen; CI = confidence interval, G = Grade.

^a^Percentages are calculated based on the number of patients with available data for each variable.

a Missing data: adjuvant (*n* = 7), body mass index (*n *= 21), CA19-9 (*n *= 126), CA19-9 postinduction (*n *= 287), CEA (*n *= 1078), CEA postinduction (*n *= 1510), chemotherapy switch (*n *= 16), cycles (*n *= 7), differentiation (*n *= 119), location (*n *= 5), lymphovascular (*n *= 39), margin (*n *= 93), metastatic disease (*n *= 3), perineural (*n *= 30), procedure (*n *= 22), radiotherapy (*n *= 32), resection (*n *= 5), size (*n *= 114), size postinduction (*n *= 330), surgical exploration (*n *= 28), tumor stage (*n *= 8), WHO (*n *= 9), ypN (*n *= 21), ypT (*n *= 145).

b ΔCA19-9: CA19-9 baseline—CA19-9 postinduction.

c ΔTumor size: tumor size baseline—tumor size postinduction.

d The number of (m)FOLFIRINOX cycles was defined as consecutive administrations, with or without dose modifications, until disease progression, switch of regimen, or transition to another treatment modality.

e Preoperative radiotherapy only.

f % of resections.

g 1-mm definition of the Royal College of Pathologists.

h Eighth edition of the American Joint Committee on Cancer Staging.

### Treatment characteristics

Patients received a median of 6 cycles (IQR = 4-8 cycles) of (m)FOLFIRINOX as initial therapy. In 357 (15.4%) patients, the FOLFIRINOX regimen was switched, primarily because of toxicity or local disease progression. Approximately, 45.3% received radiotherapy after systemic therapy. Surgical exploration with intent to resection was performed in 1125 (48.7%) patients of whom 989 (42.4%) patients underwent resection. Among patients who underwent resection, 61.5% received adjuvant chemotherapy.

### Restaging

At restaging, 175 (7.5%) patients had metastatic disease. Cross-sectional imaging showed a postinduction tumor size with a median of 30.0 mm (IQR = 23.0-44.0 mm). The median postinduction CA19-9 level was 66.7 U/mL (IQR = 23.0-239.0 U/mL), and the median postinduction carcinoembryonic antigen level was 4.2 U/mL (IQR = 2.7-6.7 U/mL).

The median overall survival after restaging was 17.2 months (95% CI = 16.1 to 18.3 months). The overall survival at 1 year after restaging was 64.0% (95% CI = 62.0% to 66.1%), 26.4% (95% CI = 24.5% to 28.4%) at 3 years, and 15.7% (95% CI = 14.0% to 17.5%) at 5 years.

### Prognostic factors for overall survival after restaging

Several baseline patient and tumor characteristics remained independent prognostic factors for overall survival after restaging: borderline resectable (HR = 1.31, 95% CI = 1.14 to 1.51) or locally advanced pancreatic adenocarcinoma (HR = 1.78, 95% CI = 1.55 to 2.05), body or tail tumor (HR = 0.79, 95% CI = 0.68 to 0.90), and baseline World Health Organization performance status of 1 vs 0 (HR = 1.18, 95% CI = 1.07 to 1.30) or a performance status of at least 2 vs 0 (HR = 1.54, 95% CI = 1.20 to 1.98). In addition, several factors available at restaging were independent factors for overall survival. Metastatic disease at restaging was the strongest factor at restaging (HR = 1.57, 95% CI = 1.31 to 1.87) (Table 2). The other 4 factors at restaging were postinduction CA19-9, ΔCA19-9, postinduction tumor size, and Δtumor size. The hazard ratio per IQR (ie, 75th vs 25th percentile) increase for log-transformed postinduction CA19-9 level was 1.47 (95% CI = 1.37 to 1.57), for log-transformed ΔCA19-9 (HR = 0.83, 95% CI = 0.77 to 0.89), for postinduction tumor size (HR = 1.22, 95% CI = 1.11 to 1.35), and for Δtumor size (HR = 0.92, 95% CI = 0.87 to 0.98). Most prognostic factors had a nonlinear association with overall survival after restaging (Figure S1).

**Table 2.** Univariable and multivariable Cox proportional hazards regression analysis of overall survival after restaging for patients with localized pancreatic adenocarcinoma

	Univariable analysis HR (95% CI)	Multivariable analysis HR (95% CI)
Risk factor^a^		
Baseline		
Sex		
Male	1 (Referent)	—
Female	0.93 (0.85 to 1.02)	
Age	1.0 (1.00 to 1.01)	—
Body mass index	0.99 (0.98 to 1.00)	—
Radiographic tumor stage		
Potentially resectable	1 (Referent)	1 (Referent)
Borderline resectable	1.43 (1.25 to 1.64)	1.31 (1.14 to 1.51)
Locally advanced	2.11 (1.86 to 2.39)	1.78 (1.55 to 2.05)
Tumor location in pancreas		
Head or uncinate	1 (Referent)	1 (Referent)
Neck or proximal body	1.03 (0.91 to 1.17)	0.90 (0.79 to 1.02)
Distal body or tail	0.87 (0.77 to 0.99)	0.79 (0.68 to 0.90)
Baseline World Health Organization performance status		
0	1 (Referent)	1 (Referent)
1	1.33 (1.21 to 1.46)	1.18 (1.07 to 1.30)
≥2	1.65 (1.29 to 2.11)	1.54 (1.20 to 1.98)
Restaging		
Metastatic disease at restaging		
No	1 (Referent)	1 (Referent)
Yes	2.87 (2.44 to 3.38)	1.57 (1.31 to 1.87)
Postinduction tumor size on Computed Tomography, mm	1.57 (1.54 to 1.59)^e^	1.22 (1.11 to 1.35)^e^
ΔTumor size^b^	0.78 (0.78 to 0.80)^e^	0.92 (0.87 to 0.98)^e^
Postinduction CA19-9^c^	1.68 (1.66 to 1.70)^e^	1.47 (1.37 to 1.57)^e^
ΔCA19-9^d^	0.69 (0.68 to 0.70)^e^	0.83 (0.77 to 0.89)^e^

Abbreviations: CA 19-9 = carbohydrate antigen 19-9; CI = confidence interval; HR = hazard ratio.

a Imputed data: body mass index (*n* = 21), CA19-9 (*n* = 126), CA19-9 postinduction (*n* = 287), location (*n* = 5), metastatic disease (*n* = 3), size (*n* = 114), size postinduction (n = 330), tumor stage (*n* = 8), and World Health Organization (*n* = 9).

b ΔTumor size in mm: baseline tumor size–postinduction tumor size.

c log-transformed.

d ΔCA19-9: log[baseline CA19-9]—log[postinduction CA19-9].

e Hazard ratio for the IQR (25th to 75th percentile).

### Clinical calculator for individualized prognosis

**Figure 1.**
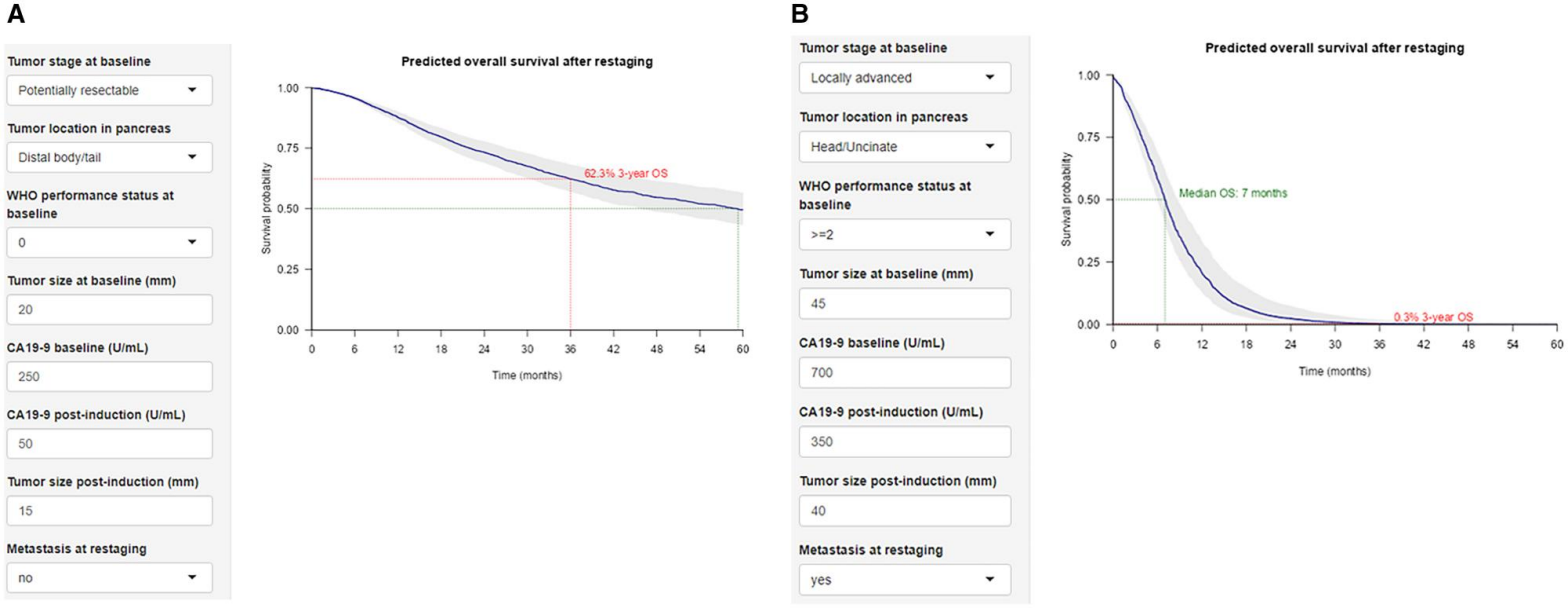
Example output of the clinical calculator. **A**) Predicted OS curve for a patient in the favorable risk group. **B**) Predicted OS curve for a patient in the very unfavorable risk group. Figure 1B illustrates the predicted survival after restaging for a patient with locally advanced pancreatic head or uncinate cancer, baseline WHO performance status of at least 2, a baseline tumor size of 45 mm, and baseline CA19-9 of 700 U/mL. After induction chemotherapy, CA19-9 decreased to 350 U/mL and tumor size to 40 mm. Metastatic disease was present at restaging. The predicted median OS after restaging was 7 months, with a predicted 3-year OS of 0.3%. Based on these characteristics, the patient is classified into the very unfavorable group. Abbreviations: CA19-9 = carbohydrate antigen 19-9; OS = overall survival; WHO = World Health Organization.

**Figure 2.**
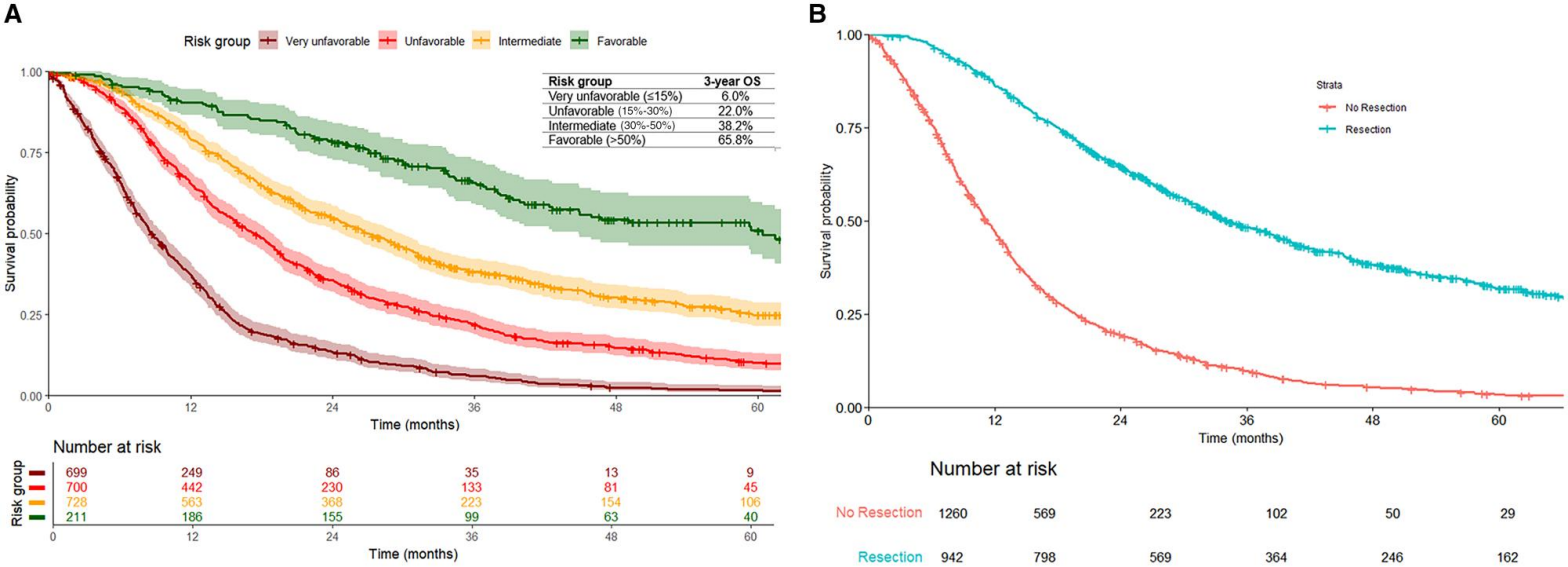
**A**) Kaplan–Meier estimates of OS after restaging stratified by model-predicted 3-year OS, pooled across multiple imputations. Risk groups were defined according to the model-predicted 3-year OS: very unfavorable (≤15%), unfavorable (15%-30%), intermediate (30%-50%), and favorable (>50%). **B**) Kaplan–Meier overall survival after restaging following induction treatment of 2202 patients with localized pancreatic adenocarcinoma treated with initial (m)FOLFIRINOX, stratified for resection. Abbreviation: OS = overall survival.

A web-based calculator was developed to predict patient survival after restaging (available at https://emmelie.shinyapps.io/taps-restaging-calculator/). The calculator generates an individual overall survival curve with predictions for the median and 3-year overall survival after restaging (Figure 1A, B). Patients were stratified into 4 risk groups based on their predicted 3-year overall survival after restaging (Figure 2A). The observed 3-year overall survival after restaging was 6.0% (95% CI = 4.4% to 8.2%) in the very unfavorable group, 22.0% (95% CI = 19.0% to 25.5%) in the unfavorable group, 38.2% (95% CI = 34.7% to 42.1%) in the intermediate group, and 65.8% (95% CI = 59.3% to 73.0%) in the favorable group. The median overall survival after restaging was 8.8 months (95% CI = 8.0 to 9.8 months) in the very unfavorable group, 17.6 months (95% CI = 15.9 to 18.9 months) in the unfavorable group, 27.1 months (95% CI = 24.7 to 29.7 months) in the intermediate group, and 60.4 months (95% CI = 44.8 to 75.9 months) in the favorable group. Among patients who underwent resection, the median overall survival after restaging was 34.3 months (95% CI = 32.0 to 38.2 months), with a 5-year overall survival of 31.9% (95% CI = 28.6% to 35.5%), whereas in patients who did not undergo resection, the median overall survival was 11.2 months (95% CI = 10.6 to 12.1 months) with a 5-year overall survival of 3.5% (95% CI = 2.6% to 4.9%) (Figure 2B). In the leave-one-center-out calibration plots for the 3-year overall survival after restaging, the Harrell C-index ranged from 0.64 to 0.70, with acceptable calibration observed across the 5 centers (Figure S2).

## Discussion

This large international cohort study identified 8 patient, tumor, and treatment response characteristics as independent prognostic factors for overall survival after restaging in patients with localized pancreatic adenocarcinoma following initial treatment with neoadjuvant (m)FOLFIRINOX. The only baseline patient factor was performance status, and the 2 baseline tumor factors were radiographic tumor stage (potentially resectable, borderline resectable, locally advanced) and tumor location. The other 5 independent factors are available at restaging: metastatic disease, postinduction CA19-9, ΔCA19-9, postinduction tumor size, and Δtumor size. Based on these factors, a web-based calculator was developed to predict overall survival after restaging for individual patients and is available via pancreascalculator.com. Patients with localized pancreatic adenocarcinoma can be classified into 4 prognostic groups after restaging, with observed 3-year overall survival rates ranging from 6.0% in the very unfavorable group to 65.8% in the favorable group. Predicted overall survival after restaging for individual patients can inform patients and guide shared decision making regarding subsequent management.

Staging and restaging form the foundation of cancer patient management, serving purposes of prognostication, shared decision making regarding treatment, and outcome evaluation.^9^ In pancreatic adenocarcinoma, the conventional TNM staging system informs primarily about the size of the tumor (T1-2), the local extent beyond the pancreas (T3-4), and the presence of distant metastases.^18^^,^^19^ Staging of lymph nodes becomes available after resection but remains inaccurate on imaging.^20^^,^^21^ Restaging after systemic chemotherapy is essential to assess treatment response, revise prognosis, and guide further management. Yet current staging systems lack standardized approaches to capture disease dynamics posttherapy.^2^^,^^9^ At restaging following neoadjuvant therapy, TNM staging considers only 2 of 8 independent prognostic factors: change in tumor size and the occurrence of metastatic disease.

To the best of our knowledge, this is the first study that used a large cohort to identify independent prognostic factors at the time of restaging after systemic treatment for localized pancreatic adenocarcinoma including all patients who started with systemic treatment. A recent systematic review and meta-analysis included 92 studies investigating prognostic factors for overall survival after neoadjuvant therapy in patients with resected pancreatic adenocarcinoma.^22^ Postinduction tumor size and normalization of CA19-9 levels were identified as prognostic factors. In the present study, change in tumor size was an independent factor of postinduction tumor size, and change in CA19-9 was an independent factor in addition to postinduction (normalization) CA19-9. Javed et al.^23^ recently developed separate prognostic models after resection of pancreatic adenocarcinoma for patients who had or had not received neoadjuvant therapy. Their retrospective study included 2760 patients with localized pancreatic adenocarcinoma who underwent a resection of whom 778 (28.2%) patients received neoadjuvant chemotherapy. The prognostic factors for patients who underwent neoadjuvant therapy included nonnormalization (≥37 U/mL) of CA19-9 after neoadjuvant therapy, nodal disease (N1/2 vs N0), and treatment response (moderate or poor vs complete or near complete). Both studies are only relevant for patients who proceeded to surgical resection after systemic treatment, which was only 42% in the present cohort. The proposed clinical calculator with 8 prognostic factors in the present study is relevant for all patients at restaging after initial systemic treatment, regardless of subsequent treatment.

Several studies have focused on the prognostic significance of CA19-9 dynamics following neoadjuvant therapy in pancreatic adenocarcinoma. Newhook et al.^24^ included 166 patients with 3 CA19-9 measurements before, during, and after neoadjuvant therapy. They found that CA19-9 response characterized by sustained decline with normalization (≤35 U/mL) (type A; HR = 0.19, 95% CI = 0.08 to 0.43) or bidirectional change with eventual normalization (type B; HR = 0.13, 95% CI = 0.04 to 0.43) were associated with the most favorable outcomes compared with continuously increasing CA19-9 levels without normalization (type E). Al Abbas et al.^25^ aimed to determine a threshold for posttreatment CA19-9 in a cohort of 250 patients with localized pancreatic adenocarcinoma who underwent resection. They found that a CA19-9 reduction of at least 85% was the strongest independent predictor of survival. The present study combined CA19-9 dynamics with 6 other independent factors to predict overall survival after restaging for individual patients. Moreover, CA19-9 dynamics was modeled with both postinduction and ΔCA19-9 levels, using continuous values rather than cutoffs for superior prediction of overall survival for individual patients.

We previously validated the clinical ABC staging system within the TAPS cohort to improve prognostication at diagnosis.^5^ Because the ABC model addressed initial staging with only baseline characteristics, it did not account for treatment response. The current model extends our previous work by integrating both factors available at baseline (ie, radiographic tumor stage, performance status, and tumor location) and restaging factors (ie, development of metastasis, postinduction and change in CA19-9, and postinduction and change in tumor size). The web-based calculator generates an individual predicted overall survival curve based on these 8 independent factors available at the time of restaging. The reassessed prognosis can inform patients and guide shared decision making for subsequent management. This aligns with the principles of personalized oncology, where prognostic insights are intended to inform, rather than dictate, therapeutic decision making and support multidisciplinary discussions.^26^ Additionally, prognostic tools should complement, not replace, individualized discussions of patients’ preferences and goals.

We included survival curves of patients who did and did not undergo resection, illustrating the markedly superior survival among patients who underwent a resection. However, this figure should be interpreted for descriptive purposes only. The observed difference in overall survival cannot be attributed solely to the effect of resection, as these cohorts markedly differ at the time of restaging. The apparent survival benefit may be partially or even entirely explained by favorable factors at restaging and unmeasured or unknown factors. The TAPS study cohort is therefore less appropriate to study treatment effects and predictive factors for treatment response. Confounding by indication and immortal time bias hamper the assessment of, for example, the overall survival benefit of surgical resection for an individual patient. Treatment effects and predictive factors are better studied in randomized controlled trials.

This study has several limitations. First, performance status was only documented at baseline, precluding assessment of functional decline or improvement at restaging. Regardless, baseline performance status remained an independent factor for overall survival after restaging. Second, missing data affected several variables, necessitating multiple imputation. Although this approach mitigates data loss compared with complete case analysis and imputation never involved more than 12% of patients, it may have introduced some bias. Third, treatment strategies and restaging protocols varied across participating centers and over time. This heterogeneity may explain suboptimal model calibration but enhanced generalizability. Fourth, our model is based on data from 5 high-volume expert centers. This may limit generalizability beyond expert centers. Fifth, data on sociodemographic factors, including race and ethnicity and insurance status, were not available in the TAPS cohort.

In conclusion, restaging patients with localized pancreatic adenocarcinoma after neoadjuvant therapy can be guided by a model including 8 readily available patient, tumor, and treatment response characteristics. The web-based calculator can be used by clinicians to inform patients and guide subsequent treatment decisions.

## Supplementary Material

djag024_Supplementary_Data

## Data Availability

The data that support the findings of this study are available from the corresponding author, B. Groot Koerkamp, upon reasonable request.
