# Supplementary material for: Restaging after neoadjuvant FOLFIRINOX for localized pancreatic cancer: a clinical calculator from the Trans-Atlantic Pancreatic Surgery consortium
Source: J Natl Cancer Inst. 2026 Mar 17;118(6):1108–14. doi: 10.1093/jnci/djag024 (PMC13247336; doi:10.1093/jnci/djag024)
Supplement: djag024_Supplementary_Data [file djag024_supplementary_data.zip › Dekker - Supplementary material.docx]

**Supplementary Material**

**Table of Contents**

1. Figure S1 page 2
2. Figure S2 page 3
3. Figure S3 page 4

**Figure S1.** Modeled associations of clinical variables for OS after restaging using a multivariable Cox model with restricted cubic splines.

**
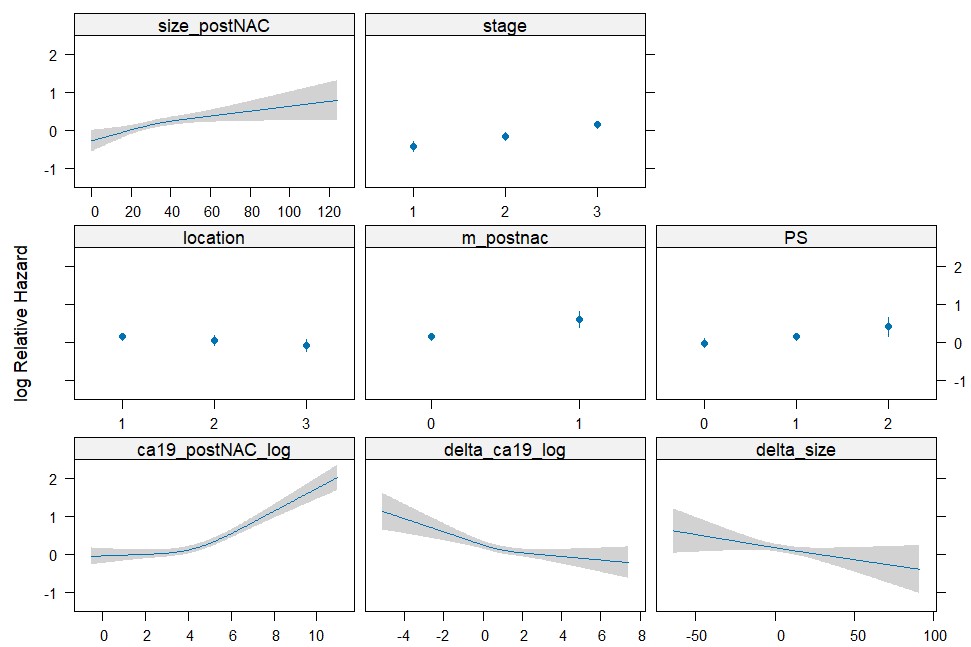
**

Abbreviations: size_postNAC= post-induction tumor size; stage= tumor stage (1=potentially resectable; 2=borderline resectable; 3= locally advanced); PS = baseline WHO performance status (0=0; 1=1; 2= ≥2); location = tumor location (1= Head/uncinate; 2=Neck/proximal body; 3=Distal body/tail); m_postnac= metastasis at restaging (0=no; 1=yes); ca19_postNAC_log= log-transformed post-induction CA19-9 level; delta_ca19_log= log[Baseline CA19-9] – log[post-induction CA19-9l]; delta_size= baseline tumor size – post-induction tumor size.

**Figure S2.** Leave-One-Center-Out Cross-Validation. Calibration Plots with Harrell’s C-Index for Each Center.


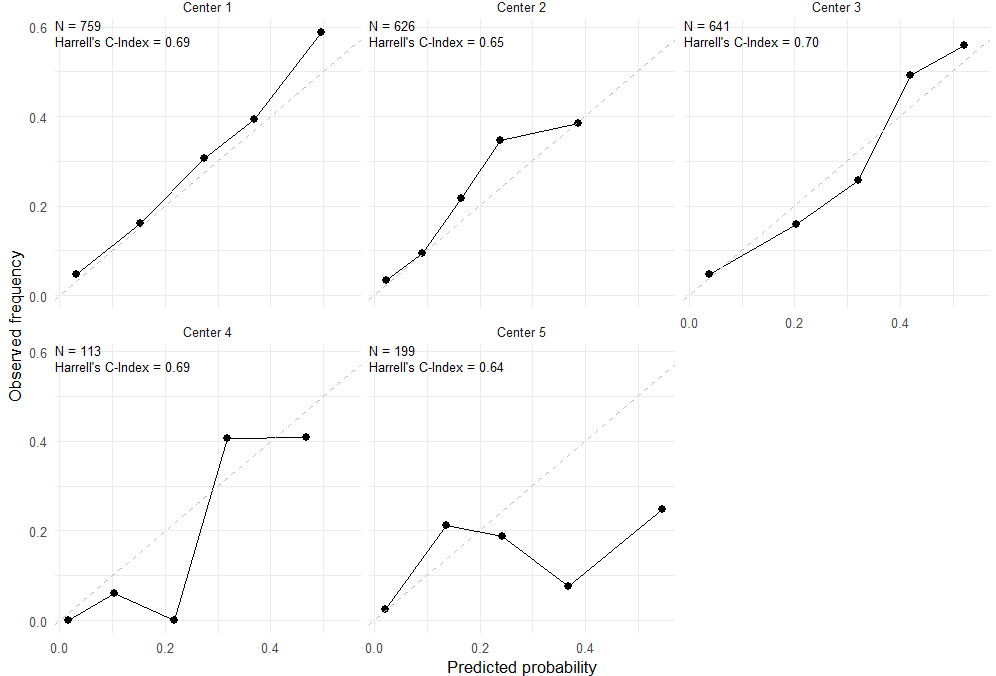


*N* indicates the number of patients included from each center.

**Figure S3.** Relative prognostic importance of predictors for overall survival at restaging.


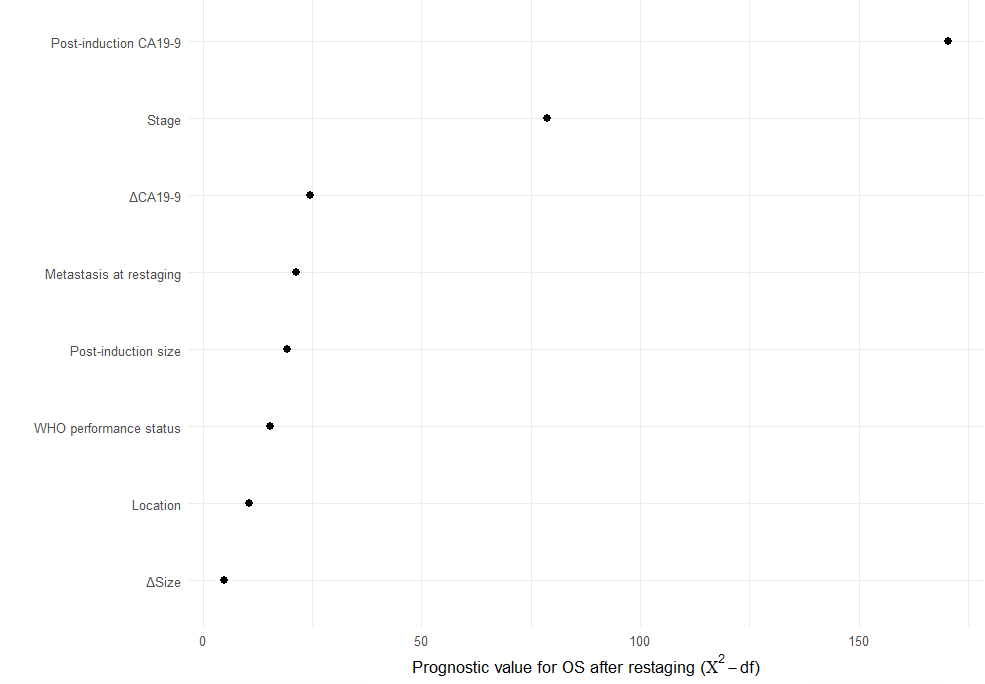


∆CA19-9 = log[Baseline CA19-9] – log[post-induction CA19-9l]; ∆Size= baseline tumor size – post-induction tumor size.
